# Biomarkers in kidney and heart disease

**DOI:** 10.1186/1471-2210-11-S1-O4

**Published:** 2011-08-01

**Authors:** Alan S Maisel

**Affiliations:** 1 VA San Diego Healthcare System, Cardiology Section, San Diego, USA

## 

Complex patients present with numerous risk factors and disease states. Clinical uses of biomarkers include diagnosis (establishing or excluding the cause of undifferentiated symptoms, understanding specific pathogenic mechanisms, or identifying concomitant conditions), risk stratification (assessing future risk of adverse outcomes or monitoring risk of adverse events), screening, and guiding therapy. Plasma natriuretic peptide are released from the heart in response to volume overload, as well as serving as markers of elevated filling pressures, a finding that in many cases would otherwise only be detectable with invasive testing. Therefore, the use of natriuretic peptides is an important adjunct to echocardiography in many circumstances. Just as BNP levels can be considered the arbitrator of CHF, cardiac troponins (TnI/TnT) are decisive for myocardial necrosis. The introduction of novel assays with even higher clinical sensitivity, detecting troponin levels at nanogram quantities, suggest even earlier diagnosis of acute myocardial infarction and identification of patients at substantial risk post-infarction. NGAL is one of the earliest and most robustly induced genes and proteins in the kidney after ischemic or nephrotoxic injury. Elevations are detectable within hours of acute kidney injury (AKI); whereas corresponding creatinine elevations lag 1 to 3 days behind.

